# Resonance assignment of coiled-coil 3 (CC3) domain of human STIM1

**DOI:** 10.1007/s12104-021-10042-7

**Published:** 2021-08-21

**Authors:** Agrim Gupta, Christian Manuel Kitzler, Petr Rathner, Marc Fahrner, Herwig Grabmayr, Adriana Rathner, Christoph Romanin, Norbert Müller

**Affiliations:** 1 Institute of Organic Chemistry, Johannes Kepler University Linz, Altenbergerstrasse 69, 4040 Linz, Austria; 2 Institute of Inorganic Chemistry, Johannes Kepler University Linz, Altenbergerstrasse 69, 4040 Linz, Austria; 3 Institute of Biophysics, Johannes Kepler University Linz, Gruberstrasse 40, 4020 Linz, Austria; 4 Faculty of Science, University of South Bohemia, Branišovská 1645/31A, 370 05 České Budějovice, Czech Republic; 5 Present Address: Institute of Analytical Chemistry, University of Vienna, Währingerstrasse 38, 1090 Vienna, Austria

**Keywords:** Calcium channel, Coiled-coil structure, Store-operated calcium entry, CRAC

## Abstract

The protein stromal interaction molecule 1 (STIM1) plays a pivotal role in mediating store-operated calcium entry (SOCE) into cells, which is essential for adaptive immunity. It acts as a calcium sensor in the endoplasmic reticulum (ER) and extends into the cytosol, where it changes from an inactive (tight) to an active (extended) oligomeric form upon calcium store depletion. NMR studies of this protein are challenging due to its membrane-spanning and aggregation properties. Therefore follow the divide-and-conquer approach, focusing on individual domains first is in order. The cytosolic part is predicted to have a large content of coiled-coil (CC) structure. We report the ^1^H, ^13^C, ^15^N chemical shift assignments of the CC3 domain. This domain is crucial for the stabilisation of the tight quiescent form of STIM1 as well as for activating the ORAI calcium channel by direct contact, in the extended active form.

**Supplementary Information:**

The online version contains supplementary material available at 10.1007/s12104-021-10042-7.

## Biological context

Calcium ions (Ca^2+^) play an essential role as second messengers in every cell. Ca^2+^ controls a variety of cellular processes including gene expression, cell division, neuronal signaling, muscle contraction, fertilization and apoptosis. Besides voltage-dependent and ligand-activated calcium channels, store-operated calcium entry (SOCE) is of major importance for cellular calcium entry (Berridge et al. 1995; Berridge et al. 2003; Putney 1986; Putney 2005). SOCE is mediated by specific ion channels that respond with high sensitivity to the depletion of the intracellular calcium stores within the endoplasmic reticulum (ER). Two key proteins form the calcium release activated calcium channel (CRAC). Orai1, which forms the Ca^2+^ channel in the plasma membrane, and STIM, which senses changes in ER-luminal Ca^2+^ concentration and controls the channel activity accordingly. The STIM protein family consists mainly of STIM1 and STIM2, with different isoforms expressed in a cell type-specific manner. STIM1 occupies the dominant role in most cell types (Roos et al. 2005; Feske et al. 2006; Prakriya et al. 2015; Fahrner et al. 2017; Grabmayr et al. 2020). In humans both STIM1 and Orai1 are associated with pathological gain-of-function (GoF) and loss-of-function (LoF) mutations. These lead to severe diseases such as severe combined immunodeficiency (SCID), tubular aggregate myopathy (TAM) and the Stormorken syndrome. The symptoms of the resulting different pathological conditions represent a clinical continuum in both GoF and LoF. Depending on the mutant form, the disease courses can be mild or even lethal (Lacruz et al. 2015; Nesin et al. 2014; Morin et al. 2014; Misceo et al. 2014; Fahrner et al. 2018; Morin et al. 2020).

In recent years, an advanced model of the store-depletion triggered STIM1 activation has been developed, giving special importance to the role of both the N-terminal ER luminal and the C-terminal cytosolic part of STIM1 (Prakriya et al. 2015; Grabmayr et al. 2020; Fahrner et al. 2020). The STIM1 N-terminus contains the calcium sensitive EF-SAM (sterile α motif) domain, which senses the luminal calcium concentration. A decrease in ER calcium concentration results in conformational change of the luminal part of STIM1, signal propagation along the transmembrane domain and triggering of a massive structural change in the C-terminal STIM1 part (Fahrner et al. 2020).

The cytosolic portion of STIM1 contains three coiled coil (CC) domains denoted as CC1, CC2, CC3 with CC1 subdivided into three alpha helices (α1, α2 and α3) (Rathner et al. 2021). The CC designation is based on bio-informatic analysis of the sequences, identifying residue repeats with high CC propensities. The basic secondary structure is α helical. But apparently, depending on the functional stage of the proteins different inter-helical CC-contacts can be formed. The designation CC-1, 2, 3 refers to segments of the structure which are α helical and separated by loops. Previous investigations imply that at least one of the functional stages is involved in an actual CC structure. The interhelical contacts may be intra- or inter-molecular in the full-length protein.

The CC2 and CC3 domains contain the main STIM/Orai1 activating region (SOAR) also known as CRAC activating domain (CAD) which enables STIM1 to couple to and activate Orai1 (Park et al. 2009; Yuan et al. 2009).

The activation mechanism of STIM1 has been intensively investigated for more than 10 years. A tight, quiescent state and an extended activated state of STIM1 have been identified. The tight state is realized by a “clamp” between CC1α1 and CC3 keeping CAD/SOAR close to the ER membrane. Upon store depletion, CC1 homomerization occurs, which elicits the release of the clamp, realizing the extended state. After a further oligomerization process potentially involving CC3, STIM1 couples to Orai1 and activates the channel (Muik et al. 2009; Fahrner et al. 2014, 2020; Rathner et al. 2021; Zhou et al. 2013).

Thus, STIM1 CC3 is an essential domain with multiple functions: In resting cells, it stabilizes the protein in the quiescent tight state. Upon activation, it engages in STIM homo-oligomerization, supporting STIM1 cluster formation. In addition, the linker region between CC3 and CC2 is an important contact domain to Orai1, essential for channel activation (Frischauf et al. 2009; Muik et al. 2009, 2011; Fahrner et al. 2014; Zhou et al. 2013). In the present study, we report the first solution NMR resonance assignment and prediction of secondary structure propensities of the monomeric CC3 domain of wild-type human STIM1.

## Methods and experiments

### Protein expression and purification

The genetic information of the human STIM1 coiled-coil3 (CC3) fragment (amino acids 388–430) was amplified by PCR and cloned into a pGEX 4T-1 vector using the Xho1 and BamH1 restriction sites. The pGEX 4T-1 vector includes a GST tag along with a thrombin cleavage site. The vector was transformed into competent BL21 *E. coli* cells using the standard heat shock method. Transformed cells were cultivated in LB medium (ampicillin, 100 mg/L) overnight at 37 °C. The bacterial stock was used to inoculate 250 mL of LB medium (1:100 dilution), the culture was further cultivated at 37 °C until optical density (OD at 580 nm) reached 0.8. The cells were gently centrifuged (2000 g, 25 °C, 20 min) and resuspended in M9 minimal medium with (^15^NH_4_)_2_SO_4_ (1.5 g/L) and ^13^C_6_-glucose (3 g/L) as sole nitrogen and carbon sources, respectively and enriched with BME vitamins (Sigma Aldrich). The culture was incubated at 37 °C for another 30 min and then isopropyl β-D-1-thiogalactopyranoside (IPTG) (final conc. 1 mM) was added and the temperature lowered to 28 °C before overnight cultivation. After centrifugation, the cell pellet was suspended in 20 mM Tris-HCl, 100 mM NaCl, 5 mM β-mercaptoethanol (βME), 10 % Glycerol, 0.1 % Triton X-100, pH 7.5 adding 4-(2-Aminoethyl)benzenesulfonyl fluoride hydrochloride (AEBSF) (36 µg/mL, neoLab) as protease inhibitor. After sonication on ice (6 cycles of 1 min), an existing purification protocol (Rathner et al. 2018) was slightly modified in order to increase the yield of soluble CC3 protein fragment. The homogeneity of the fragment was monitored by SDS-PAGE and the protein sample was concentrated to 0.5 mM using Amicon® ultra centrifugal filters (Merck) with 3 kDa cut-off and stored at 4 °C.

### NMR spectroscopy

The purified protein samples were swapped to 20 mM Bis–Tris, 10 % v/v D_2_O, pH 6.0 and 550 µL (protein concentration 0.5 mM) transferred to 5 mm NMR tubes (Wilmad 535PPT). Following previous studies, 2,2,2-trifluoroethanol (TFE) buffer additive at various concentrations was tested to suppress aggregation and obtain sufficient resolution in ^1^H–^15^N HSQC spectra (Rathner et al. 2018; Stathopulos et al. 2013). The final resonance assignment was carried out with the addition of 17.5 % v/v TFE at 310 K by the combination of common 2D and 3D experiments: ^1^H–^15^N HSQC, ^1^H–^13^C HSQC, HNCACB, HN(CA)CO, HNCO, HNCA, H(CCO)NH and CC(CO)NH (Montelione et al. 1992; Grzesiek and Bax 1993; Sattler et al. 1999). All the NMR experiments were performed on a 700 MHz Bruker Avance III spectrometer equipped with a TCI cryogenically cooled (20 K) probe and processed by Topspin^®^ software (version 3.6). Backbone dihedral angles and secondary structure propensities were predicted using TALOS-N (Shen and Bax 2013). Additionally, a structure prediction with CS-Rosetta software was performed (Shen et al. 2008).

### CD Spectroscopy

The CD spectra were obtained using the Jasco J-810 spectropolarimeter by averaging of three measurement runs (180–280 nm, 10 nm/min scanning speed) at 20 °C using a quartz cuvette with 0.1 cm path length at sample concentration of 50 µM and processed with Jasco spectra manager (v. 2.14). In order to evaluate the impact of TFE on the CC3 secondary structure, the spectra were measured in 20 mM Bis-Tris, pH 6.0 with and without addition of 17.5 % v/v TFE. The coiled-coil index was determined from the global and local minima at 208 nm and 220 nm. The ratio r = [CD]_222_/[CD]_208_ ≥ 1 indicates a coiled coil motif whereas r ≤ 0.86 indicates an isolated α helix (Greenfield 2006).

### Dynamic light scattering

Aggregation properties of isolated STIM1 CC3 were probed by dynamic light scattering (DLS) using a Zetasizer Nano ZSP 2 (Malvern). Measurements were carried out in 15 mm centre height UV-micro cuvettes and were performed by averaging 6 runs (with automatic optimized repetitions of 10 s measurements) for both CC3 in 20 mM Bis-Tris and CC3 in 20 mM Bis-Tris, 17.5 % v/v TFE at 20 °C. The analysis was performed using the Zetasizer Software version 8.01 (Malvern).

### Extent of assignments and data deposition

^1^H–^1^^5^N Heteronuclear single quantum coherence spectroscopy (HSQC) and transverse relaxation-optimized spectroscopy (TROSY-HSQC) experiments were first run at 298 K in 20 mM Bis–Tris at pH 6.0. Extensive signal overlap presumably due to coiled-coil aggregation of the domain (see coiled-coil formation prediction in Supplementary Data Fig. S1), and sample instability made it impossible to find a sufficient number of assignable resonances. In order to reduce peak overlap and increase resolution in the HSQC-type spectra, measurements at lower protein concentration as well as pH screening including various buffer components (NaCl, HEPES, etc.) were carried out. However, there was no significant improvement in the spectral resolution as documented in the Supplementary Data Fig. S2 a). Only with the increasing concentration of TFE the number of observable resonances increased (Fig. S2 b–e). Finally, the aggregation tendency of the CC3 domain was overcome upon addition of 17.5 % v/v 2,2,2-TFE at 310 K. These conditions had successfully been used in a previous NMR study of STIM1 coiled coil fragments (Stathopulos et al. 2013), where TFE was shown to be an excellent additive to dissociate strongly aggregating coiled coil polypeptides. While preserving the tertiary structure intact, it enables protein-protein interaction studies in solution without high-order oligomerization. From previous experimental NMR in vitro and in vivo studies it appears that in presence of ionic detergents or organic solvents (TFE) stable monodisperse solutions of the STIM cytosolic fragments are observed which exhibit close structural similarities to the active form of functional full length STIM1 (Stathopulos et al. 2013; Rathner et al. 2021). In the absence of TFE the isolated CC3 structure apparently “saturates” the missing intramolecular coiled-oil contacts within full-length STIM1 by homo-oligomerization. Thus the use of an additive for the CC3 fragment serves the purpose of avoiding CC3–CC3 coiled-coil interactions, because in full-length STIM1 the CC contacts occur between different domains.

**Fig. 1:**
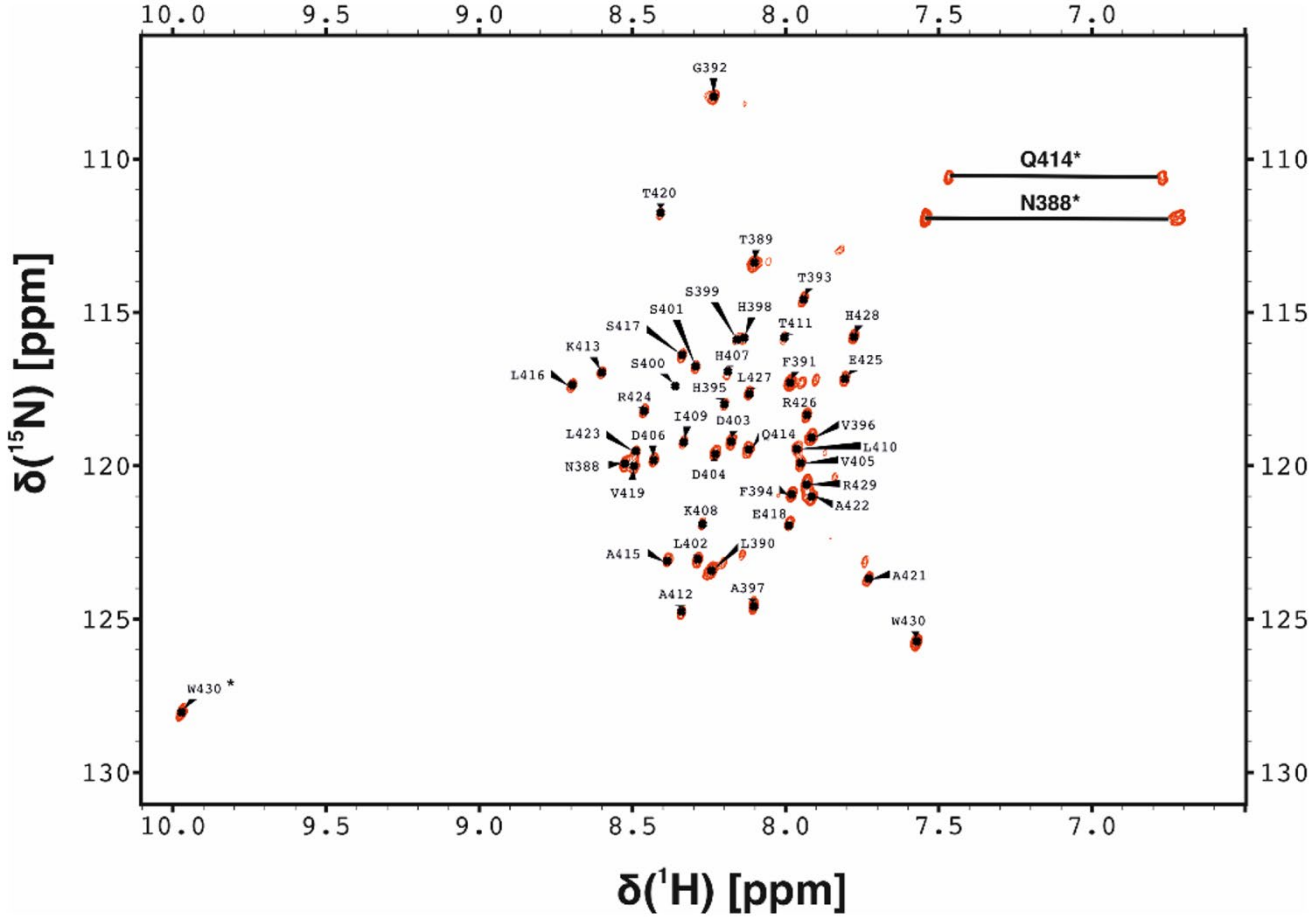
Assigned ^1^H–^15^ N HSQC spectrum of human wild-type STIM1 CC3 acquired in 20 mM Bis–Tris, 17.5 % TFE, pH 6.0 at 310 K at a protein concentration of 0.5 mM. The peaks labelled with asterisks (*) originate from side-chain N–H correlations of Trp430, Asn388 and Gln414

Under the final conditions the 2D ^1^H–^15^N HSQC spectrum shows well resolved resonances for all 43 residues (Fig. 1). We have assigned 99.5 % of the backbone resonances which include all of ^1^HN, ^15^N, ^13^Cα, ^13^CO and 97.7 % of the Hα resonances. Side-chain assignment includes fully assigned resonances of ^13^Cβ followed by 95.4 % of Hβ, 90.3 % of Cγ, 80.9 % of Hγ, 35.48 % of Cδ, 40 % of Hδ and 24 % of Hε. The aromatic residues have not been assigned yet. These assignments serve as a basis for future interaction studies between different domains within STIM1. The assigned chemical shifts have been deposited into BioMagResBank (http://www.bmrb.wisc.edu) with accession number 50683.

### Structure prediction

**Fig. 2:**
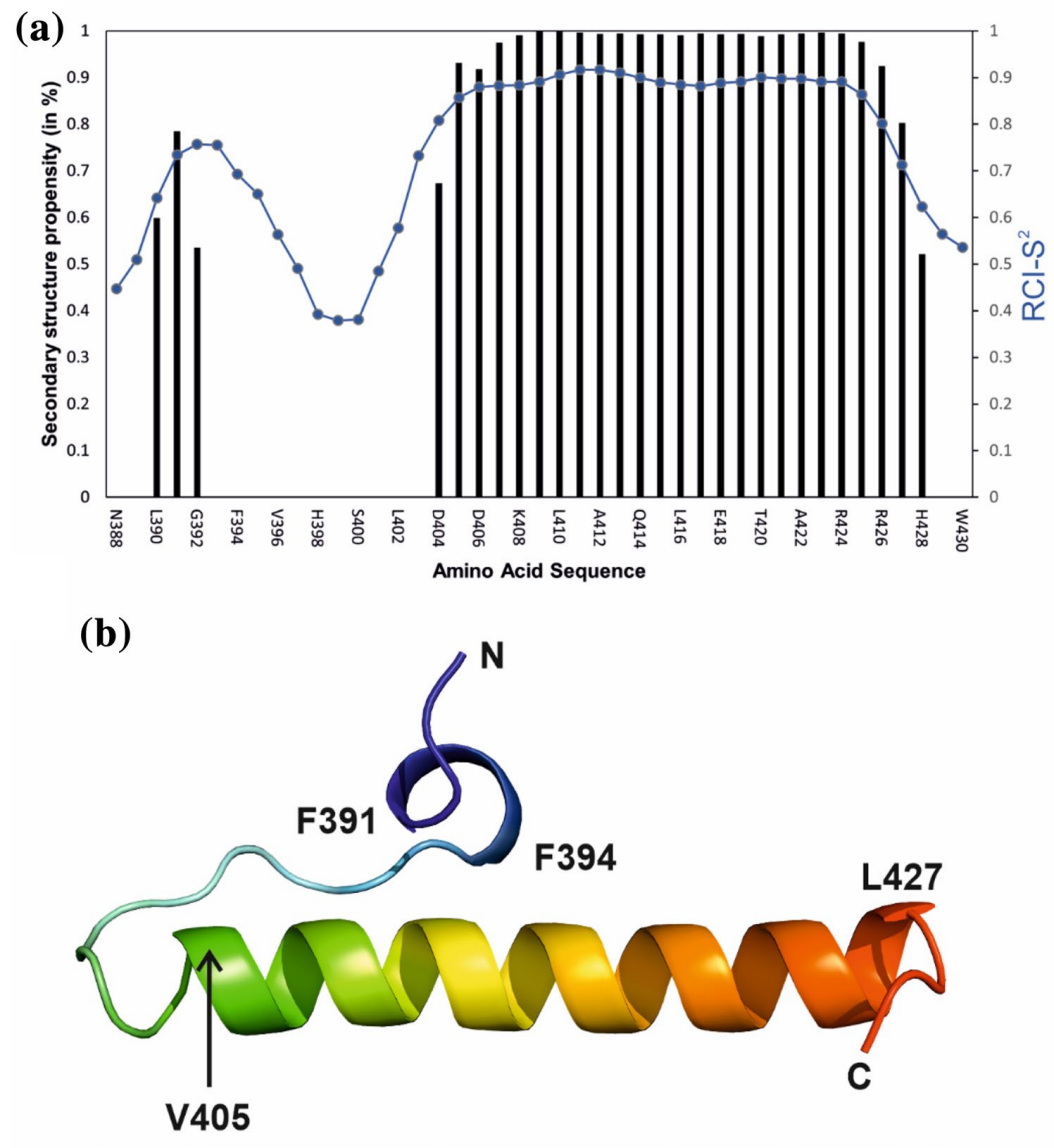
Secondary structure prediction for STIM1 CC3 **a** Results from TALOS-N (Shen and Bax 2013). Blue lines represent the order parameter RCI-S^2^ predicted from the chemical shifts. Black bars indicate secondary structure propensities for each residue. **b** Three-dimensional structural model of the monomeric STIM1 CC3 fragment from CS-Rosetta

From the chemical shift data of ^13^Cα, ^13^Cβ, ^13^CO, ^1^Hα, ^1^HN and ^15^N shifts the secondary structure propensities using TALOS-N web-based software were obtained (Fig. 2a; Shen and Bax 2013). The chemical shift data were used for the generation of 10,000 structures using CS-Rosetta (Shen et al. 2008). The structure with the smallest ratio of lowest energy and RMSD was selected and is displayed in Fig. 2b. The main structural features are a short (single turn) α-helix at the N-terminus followed by approximately 10 amino acids in a disordered loop region (H395 to D404) and a longer (ca. 6 turn) α-helix (V405 to L427).

### CD spectroscopy

**Fig. 3:**
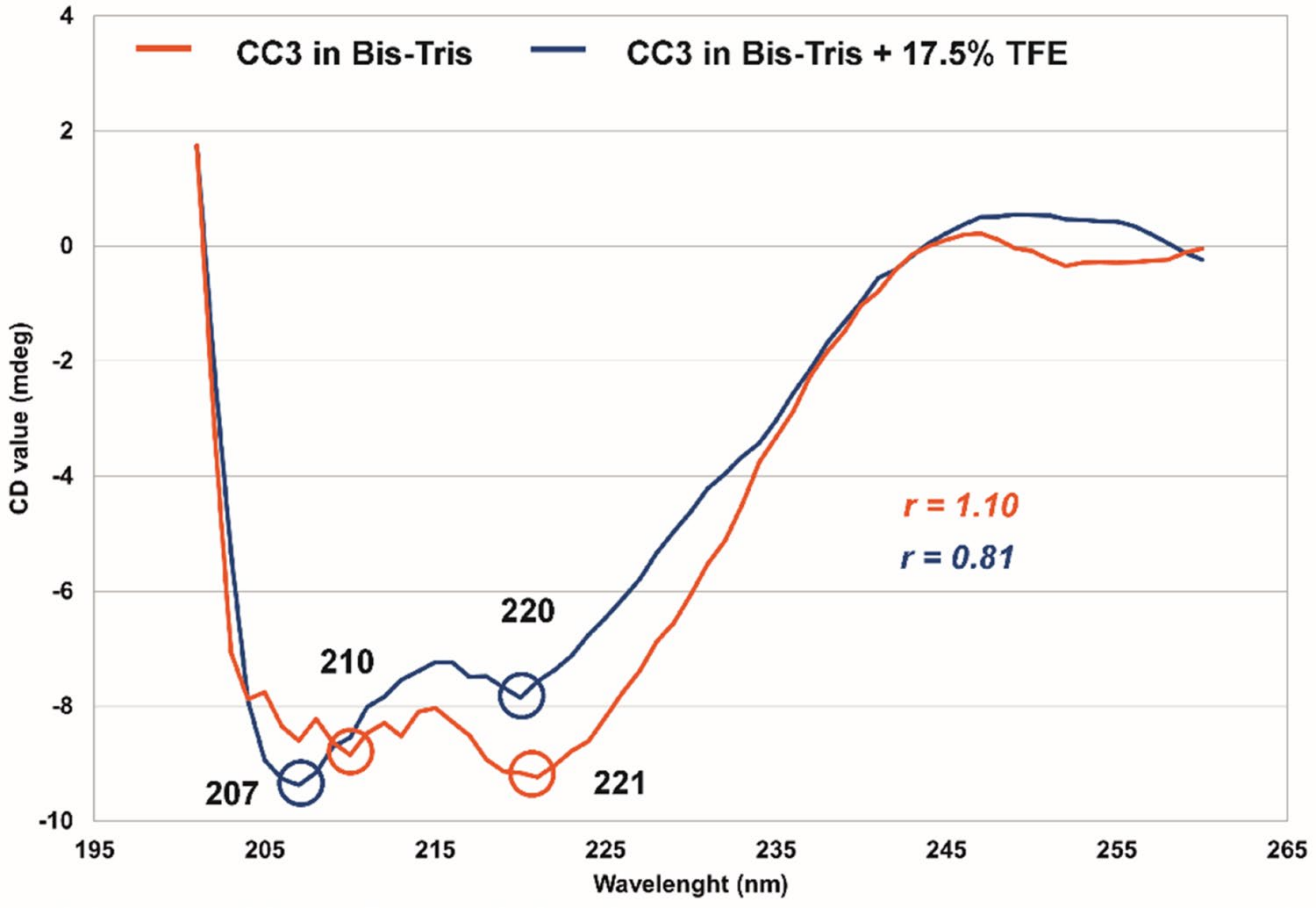
CD spectra of 0.5 mM STIM1 CC3 WT in absence (red) and presence of (blue) 17.5 % v/v TFE at 20 °C

The CD profile (Fig. 3) of STIM1 CC3 corresponds to a protein with high α helical content. The ratio *r* of [CD]_222nm_/[CD]_208nm_ without the TFE is 1.10, which is clear evidence that STIM1 CC3 forms predominantly coiled-coil oligomers. By contrast, in the presence of 2,2,2-TFE, the α helical CD profile remained but the [CD]_222nm_/[CD]_208nm_ ratio was reduced to 0.81 which is characteristic of isolated α helix (Greenfield 2006).

### Dynamic light scattering

DLS scans of free CC3 in 20 mM Bis-Tris in the absence of TFE showed existence of higher order oligomers (Fig. S3 a). The population was classified as polydisperse with the major mass maximum around 443 kDa. The calculated radius was 7.84 nm at 20 °C for the aggregated sample in the absence of TFE. Repetition of the same experiment with addition of 17.5% v/v TFE resulted in much smoother correlation decay curve. However, the exact determination of the protein size and weight was hindered by the presence of the TFE co-solvent itself (Fig. S3 b) (Gast and Modler 2007).

## Conclusions

We have reported complete backbone and near-complete side chain NMR resonance assignments of the human wild-type STIM1 CC3 domain. Based on these data, we obtained a prediction of its secondary structure propensities along with its tertiary structure model in the presence of 2,2,2-trifluoroethanol. As expected from the coiled-coil characteristics of the primary sequence, the monomeric isolated STIM1 CC3 domain is predominantly α-helical, if the coiled-coil interactions are suppressed by a suitable co-solvent. In the monomeric state, a short disordered segment (H395 to D404) interrupts the α-helix close to the N-terminus. These data will serve as a basis for further structural and in vivo functional studies of the STIM1 activation mechanism, for which coiled-coil interaction moieties in the CC3 domain are crucial (Fahrner et al. 2018).

## Supplementary Information

Below is the link to the electronic supplementary material.

Supplementary file1 (DOCX 908 kb)

## Data Availability

The chemical shift values for the ^1^H, ^13^C and ^15^N resonances have been deposited in the BioMagResBank (https://www.bmrb.wisc.edu) under Accession Number 50683.
