# Supplementary material for: Resonance assignment of coiled-coil 3 (CC3) domain of human STIM1
Source: Biomol NMR Assign. 2021 Aug 21;15(2):433–9. doi: 10.1007/s12104-021-10042-7 (PMC8481183; doi:10.1007/s12104-021-10042-7)
Supplement: Supplementary file 1 — Supplementary file1 (DOCX 908 kb) [file 12104_2021_10042_MOESM1_ESM.docx]

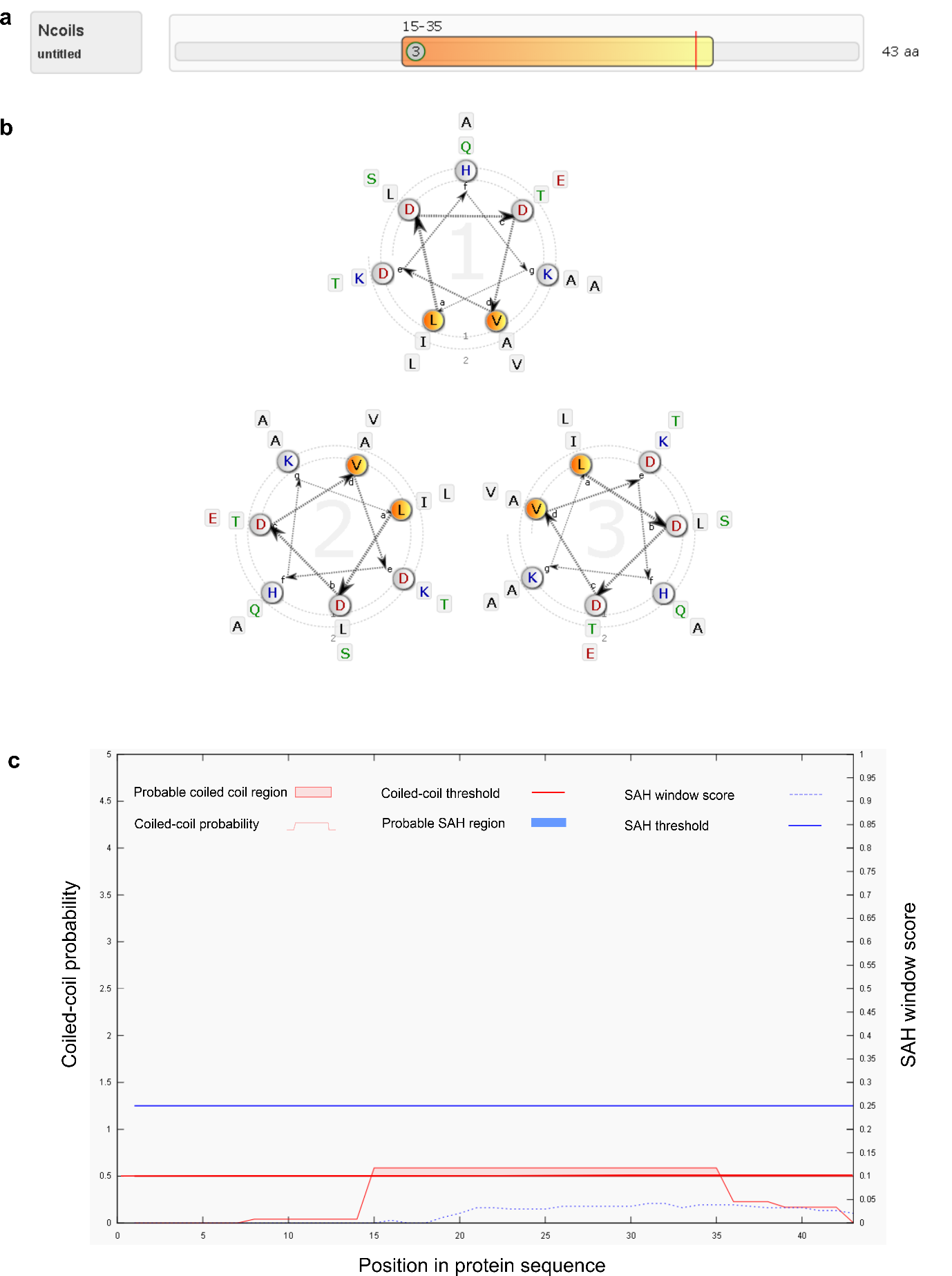


**Fig. S1** Coiled-coil prediction by “Wagga-wagga” webserver. **a** Overview of the STIM1 CC3 domain: The prediction for coiled-coil regions was performed with the tool ‘Ncoils’ starting from left to right of the sequence. The highlighted box at residues 15-35 shows a coiled-coil region. The oligomerization state of the region has been classified as a trimer. **b** Helical wheel view: The potential interactions between residues inside a predicted coiled-coil for STIM1 CC3. **c** Coiled-coil and SAH prediction plot: The coiled-coil and single α helix (SAH) probability score of STIM1 CC3. SAH-score predicted for CC3 is 0.0357. In general, high SAH-score (values >= 0.25) is a strong indicator for a single & alpha-helix instead of a predicted coiled-coil (Simm et al. 2015).


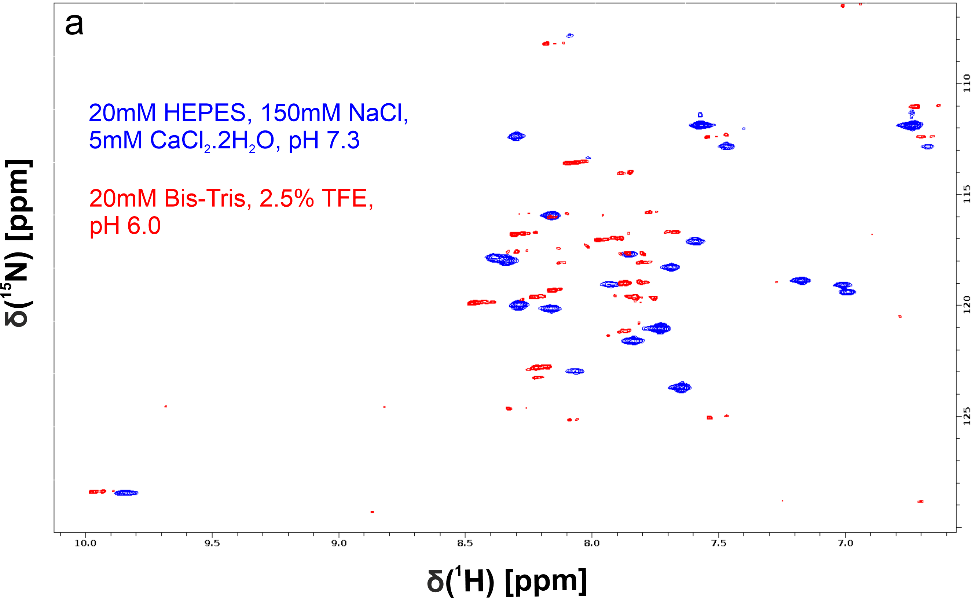


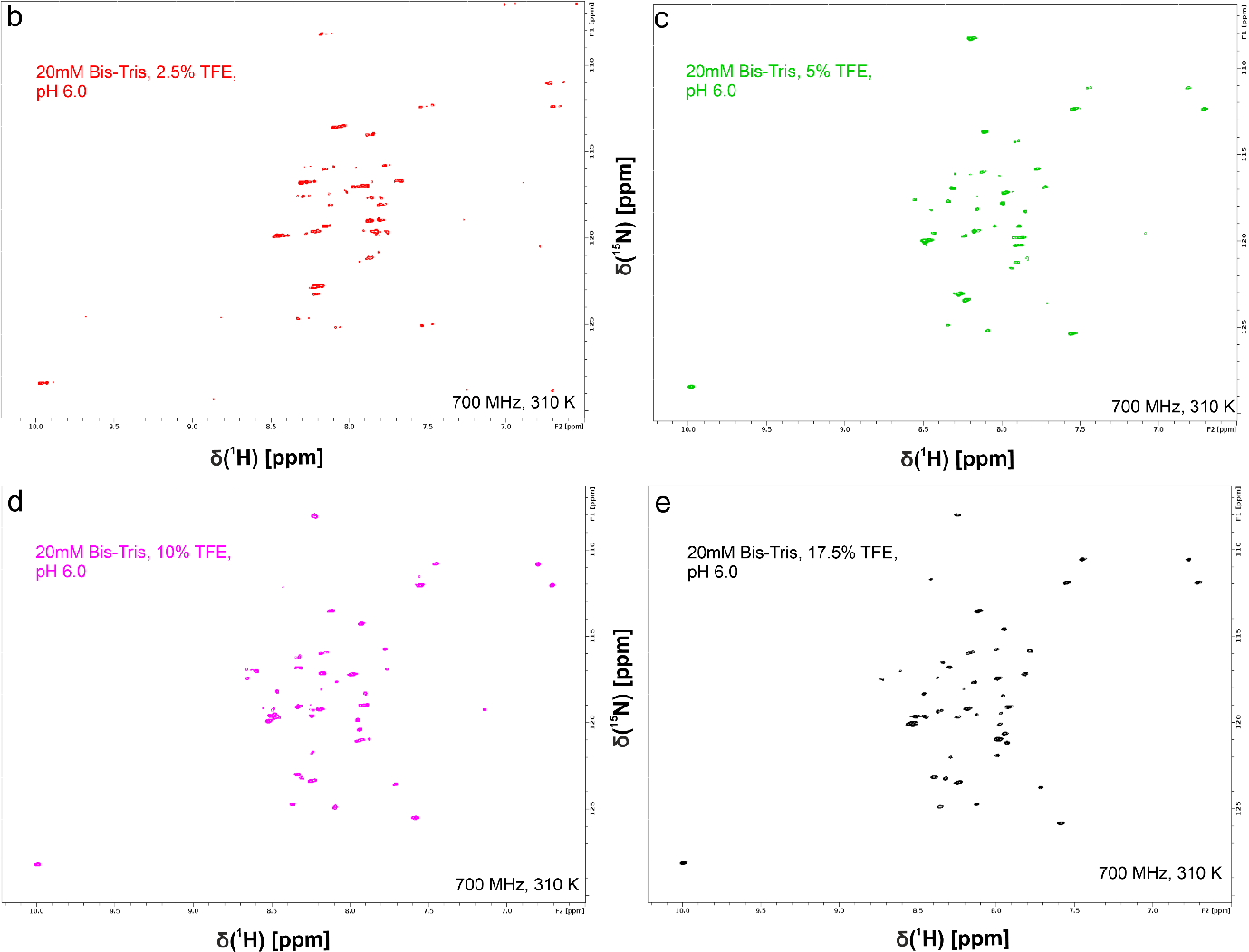


**Fig. S2** Buffer optimization for NMR studies of WT CC3. **a** The ^1^H-^15^N HSQC of CC3 in 20mM HEPES, 150mM NaCl, 5mM CaCl_2_.2H_2_O, pH 7.3 (blue cross peaks) only shows limited number of signals. WT CC3 in 20mM Bis-Tris, pH 6.0 with small amount of TFE (2.5% v/v) did not significantly improve the number of signals (red cross peaks). **b** ^1^H-^15^N HSQC spectra of WT CC3 with 2.5% TFE. **c** ^1^H-^15^N HSQC spectra of WT CC3 with 5% TFE. **d** ^1^H-^15^N HSQC spectra of WT CC3 with 10% TFE. **e** ^1^H-^15^N HSQC spectra of WT CC3 with 17.5% TFE.


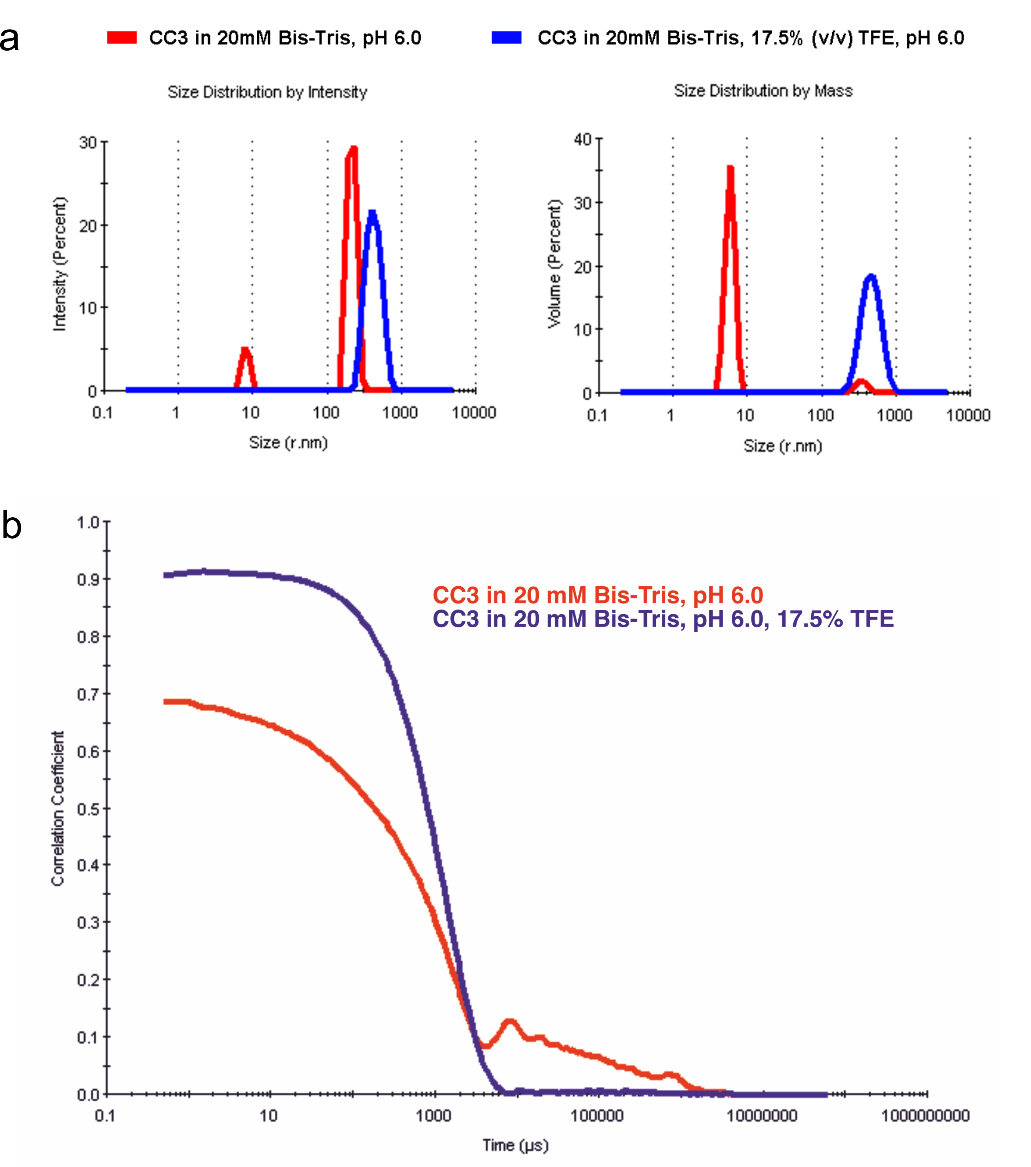


**Fig. S3** Dynamic light scattering (DLS) of STIM1 CC3. **a** Distribution of size by intensity (left) and mass (right). **b** Correlation curve of STIM1 CC3 in 20mM Bis-Tris (red) and after the addition of 17.5% TFE (blue) at 20°C.

**Reference:**Simm D, Hatje K, Kollmar M (2015) Waggawagga: comparative visualization of coiled-coil predictions and detection of stable single α-helices (SAH domains). Bioinformatics 31(5): 767-769. https://doi.org/10.1093/bioinformatics/btu700
